# Left Anterolateral Medullary Compression Due to Vertebral Artery Dolichoectasia: A Case Report and Review of Literature

**DOI:** 10.7759/cureus.67361

**Published:** 2024-08-21

**Authors:** Ajina Sam, Sanjaykanth B, Karthik Krishna Ramakrishnan, Michael Antony Vikram, Arunkumar Mohanakrishnan

**Affiliations:** 1 Radiodiagnosis, Saveetha Medical College and Hospital, Saveetha Institute of Medical and Technical Sciences, Saveetha University, Chennai, IND

**Keywords:** ectasia, mri, vbd, vertebral compression syndrome, vertebrobasilar dolichoectasia

## Abstract

Vertebral artery dolichoectasia, characterized by the elongation, dilation, and tortuosity of the vertebral arteries, poses significant clinical challenges due to its potential to compress adjacent neural structures, particularly the medulla oblongata. This case report presents a 73-year-old hypertensive female with recurrent episodes of falls and transient loss of consciousness. Initial assessments including echocardiography and a four-vessel Doppler study were unremarkable, prompting further evaluation with MRI. High-resolution imaging sequences revealed significant dolichoectasia of the left vertebral artery, compressing the left anterolateral medulla. This compression disrupted vital autonomic and motor pathways, explaining the patient's symptoms. Management involved a multidisciplinary approach, incorporating conservative measures, potential endovascular intervention, and neurosurgical consultation. This case underscores the importance of advanced imaging techniques in diagnosing vertebral artery dolichoectasia and highlights the need for a comprehensive, multidisciplinary treatment strategy to optimize patient outcomes.

## Introduction

Medullary compression caused by vertebral artery dolichoectasia, though uncommon, is a noteworthy clinical concern. This vascular anomaly involves the elongation, dilation, and tortuosity of the vertebral arteries, which can compress nearby neural structures such as the brainstem. It predominantly affects elderly individuals and is often linked to conditions like chronic hypertension and atherosclerosis [1].

The medulla oblongata, forming the lower part of the brainstem, is a crucial structure that houses vital autonomic and motor pathways. Compression of the medulla can disrupt these pathways, leading to a diverse range of neurological symptoms. Specifically, compression of the left anterolateral medulla can result in dysregulation of cardiovascular and respiratory functions, as well as impaired motor coordination and balance, manifesting as recurrent falls and syncope [2].

Dolichoectasia of the vertebral arteries is frequently under-diagnosed because of its variable clinical presentations and the subtle nature of imaging findings. Nonetheless, advancements in MRI have significantly improved the detection and characterization of this condition. High-resolution imaging techniques like balanced fast-field echo (BFFE) sequences allow for detailed visualization of the neurovascular anatomy, facilitating precise identification of neurovascular conflicts [3].

In this context, the Philips Multiva 1.5T MRI system provides a comprehensive range of imaging capabilities, which are especially beneficial for evaluating vertebral artery dolichoectasia. By employing sequences such as T1-weighted imaging (T1WI), T2-weighted imaging (T2WI), fluid-attenuated inversion recovery (FLAIR), and BFFE, radiologists can acquire high-resolution images that reveal both vascular abnormalities and their effects on adjacent neural structures [4].

This report thoroughly examines the case of a 73-year-old woman with hypertension who experienced multiple falls and brief episodes of unconsciousness. Advanced imaging techniques using the Philips Multiva 1.5T MRI system were instrumental in diagnosing left anterolateral medullary compression due to vertebral artery dolichoectasia. This report aims to emphasize the importance of early and accurate diagnosis through advanced imaging modalities, which is crucial for guiding appropriate management and improving patient outcomes.

## Case presentation

A 73-year-old woman with a history of hypertension visited the Emergency Department at Saveetha Medical College and Hospital in Chennai, reporting multiple instances of falls and brief episodes of unconsciousness over the past week. Each episode, occurring three to four times, lasted a few seconds to a minute without any preceding symptoms like chest pain, palpitations, or dizziness. The patient reported no history of focal neurological deficits, visual disturbances, or speech abnormalities.

On examination, the patient’s vital signs were stable, with a blood pressure of 130/80 mmHg, heart rate of 72 beats per minute, respiratory rate of 18 breaths per minute, and oxygen saturation of 98% on room air. The patient was afebrile. The neurological examination revealed that the patient was alert and oriented to time, place, and person. Bilateral presbyacusis was noted, but other cranial nerve functions were intact, including normal extraocular movements, facial symmetry, and no dysarthria. Muscle strength was 5/5 in all extremities with normal muscle tone and no tremors or involuntary movements were observed. Sensation to light touch, pain, and vibration was normal in all extremities. Deep tendon reflexes were normal and symmetric and plantar responses were flexor bilaterally. Mild unsteadiness was observed during tandem walking, but the Romberg test was negative. A cardiovascular examination revealed normal heart sounds with no murmurs or extra heart sounds. Peripheral pulses were palpable and symmetric. The respiratory examination was unremarkable with clear breath sounds bilaterally and no wheezes or crackles. The ENT examination confirmed bilateral presbyacusis, with no other abnormalities noted.

Given the recurrent nature of the episodes and the absence of clear cardiovascular or systemic causes, a detailed neurological assessment was undertaken. The initial investigations comprised echocardiography and a four-vessel Doppler study of the neck, both of which showed normal cardiac function and no significant stenosis or abnormalities in the carotid or vertebral arteries. To exclude transient ischemic attacks (TIAs) or other structural causes, an MRI of the cervical spine and brain was conducted. The imaging protocol included T1WI for anatomical detail, T2WI for identifying pathological changes, FLAIR for detecting periventricular and cortical abnormalities, diffusion-weighted imaging (DWI) for identifying acute ischemic changes, and BFFE sequences for high-resolution imaging of neurovascular structures.

**Figure 1:**
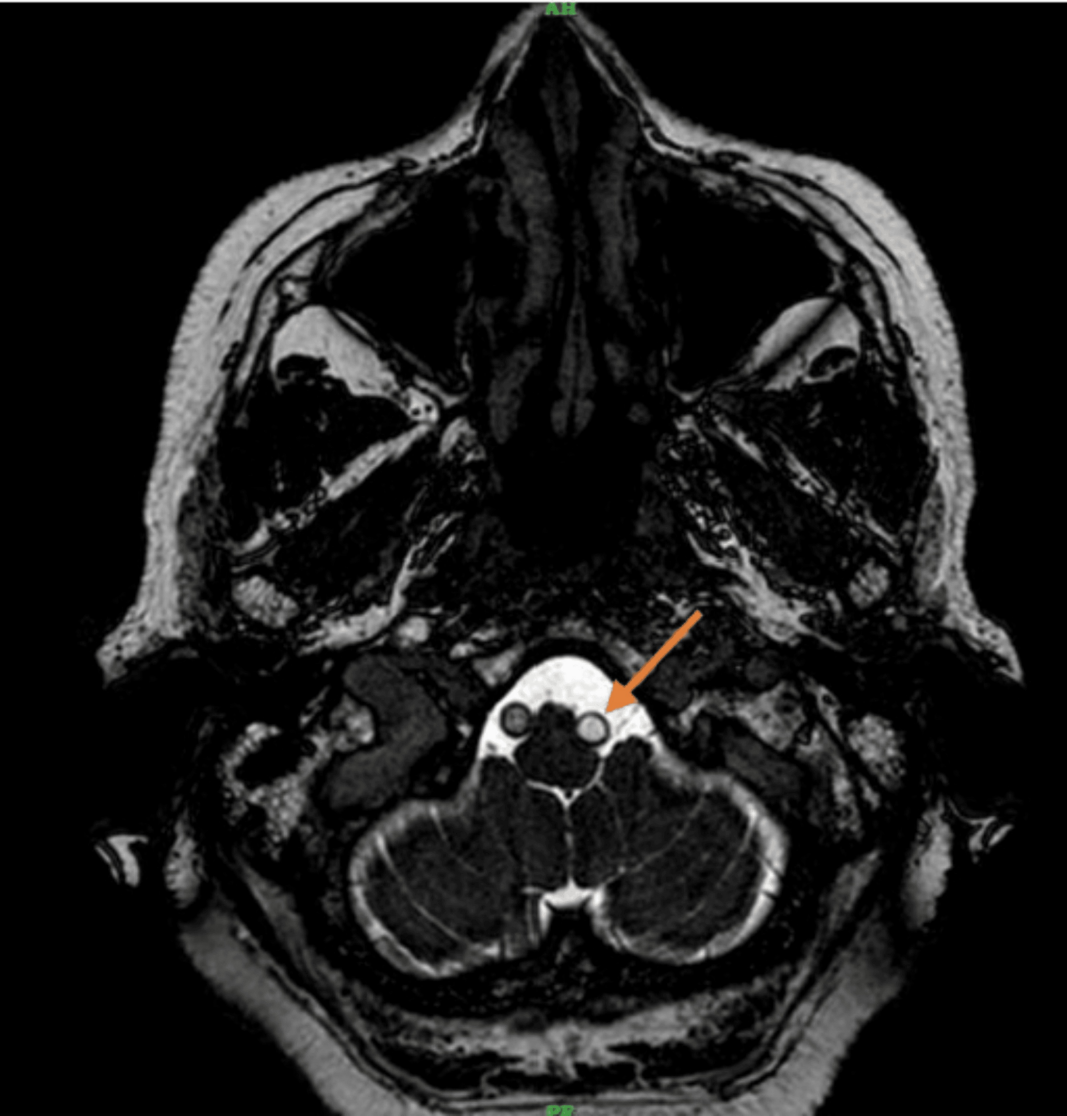
BFFE sequence of the brain-axial section showing ectatic left vertebral artery compressing the left anterolateral medulla BFFE, balanced fast-field echo

**Figure 2:**
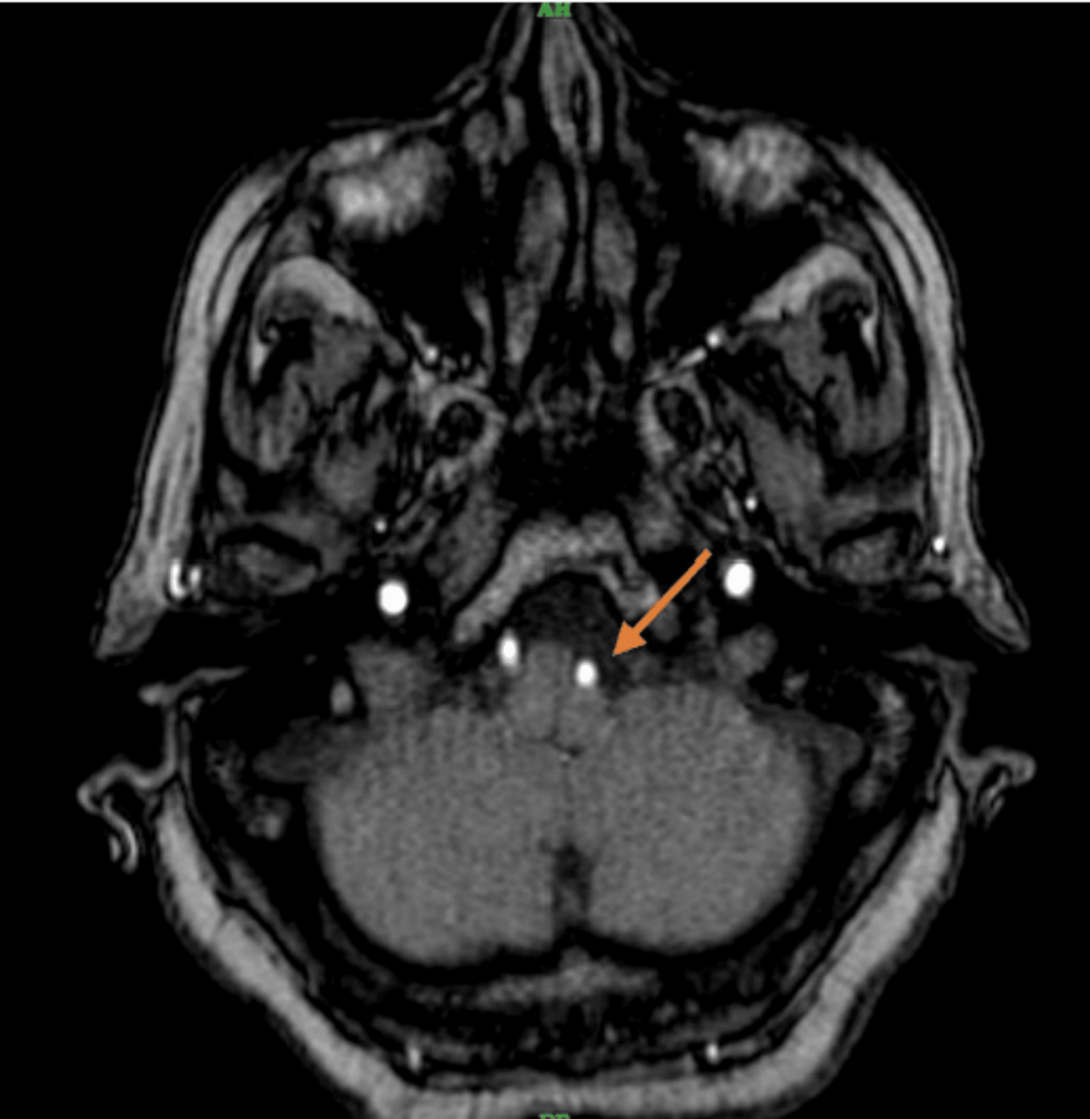
MRA brain-axial section showing ectatic left vertebral artery compressing the left anterolateral medulla

**Figure 3:**
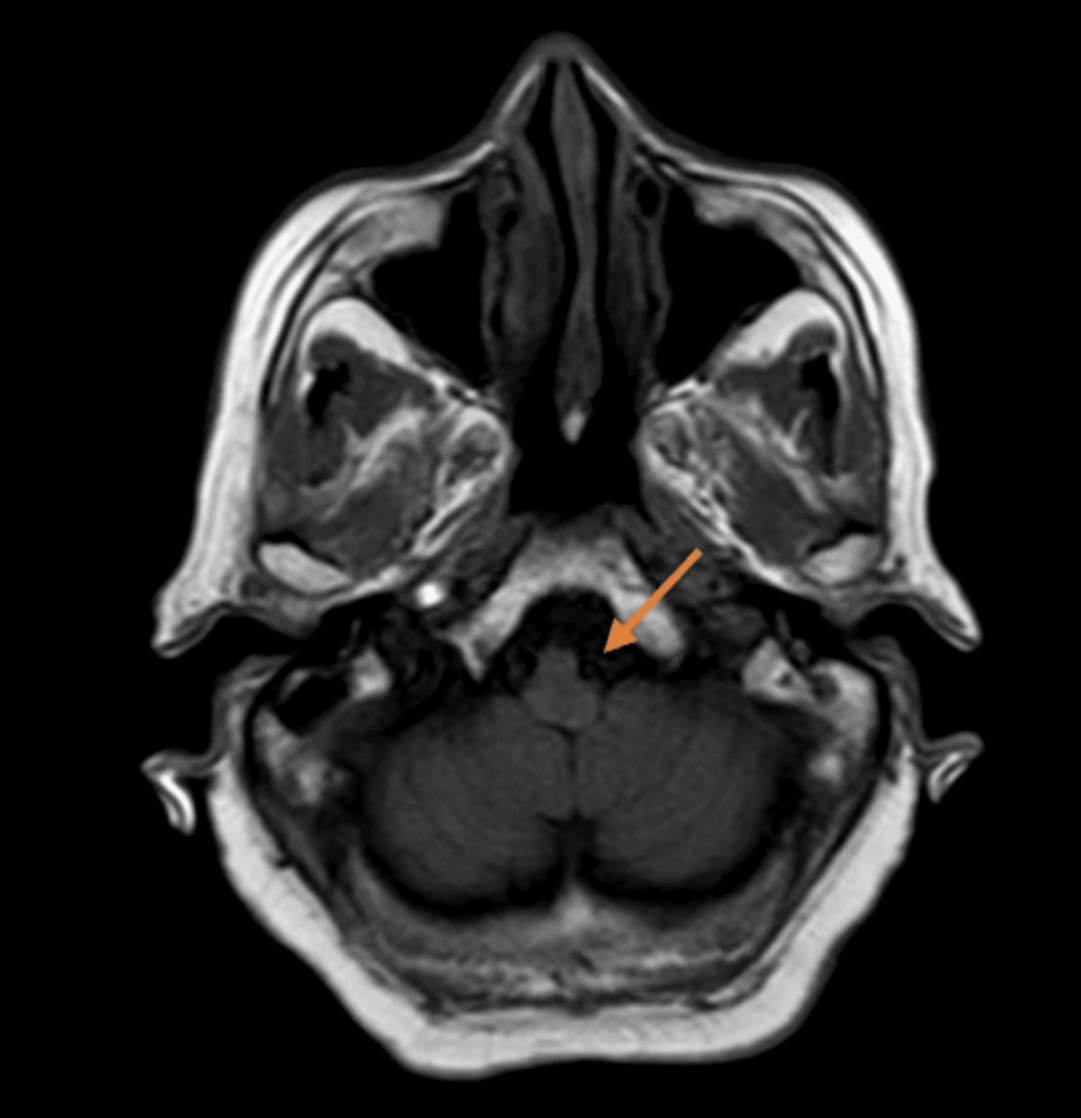
T1-weighted image of the brain-axial section showing ectatic left vertebral artery compressing the left anterolateral medulla T1WI, T1-weighted image

**Figure 4:**
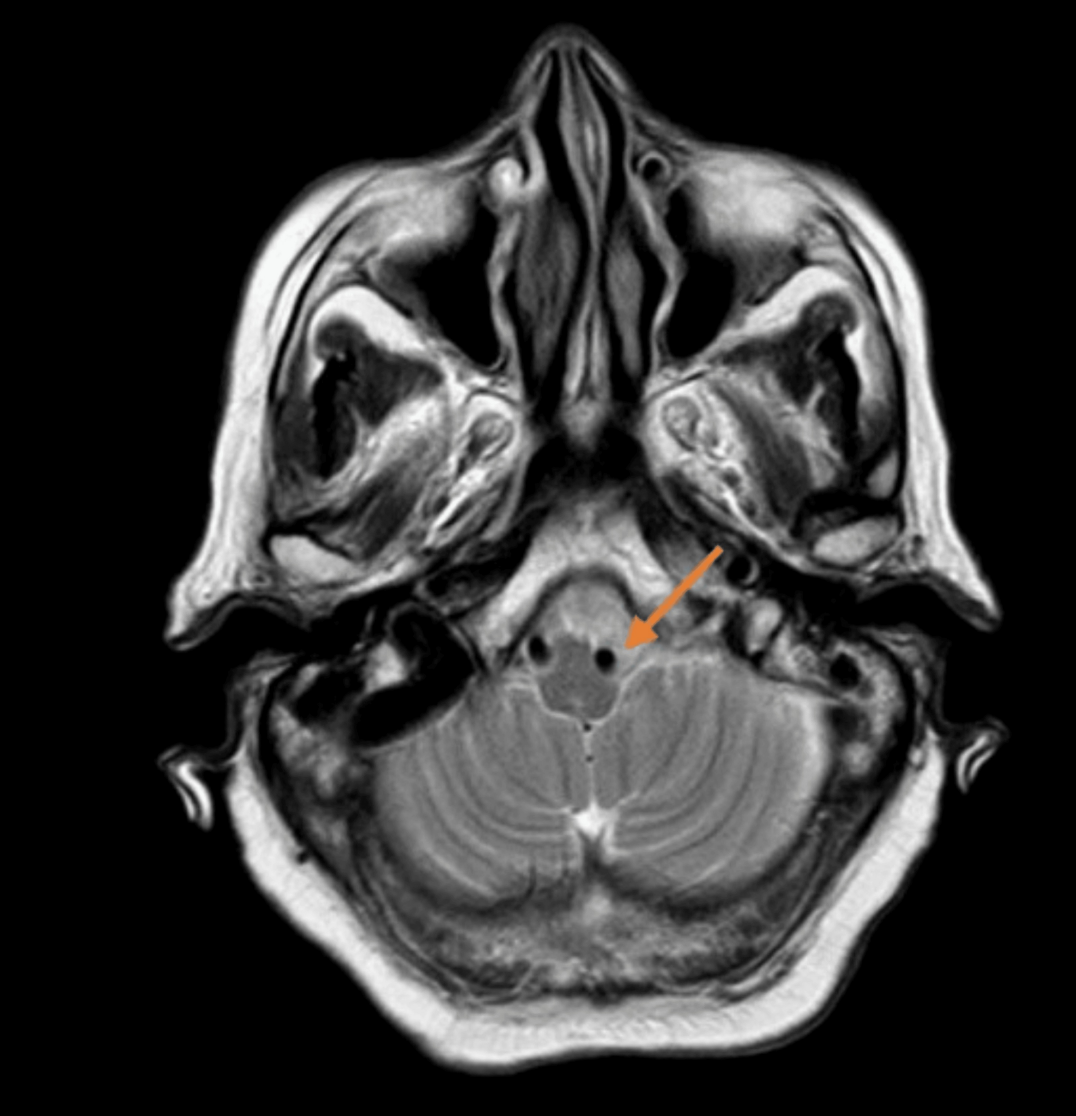
T2 weighted image of the brain-axial section showing ectatic left vertebral artery compressing the left anterolateral medulla T2WI, T2-weighted image

**Figure 5:**
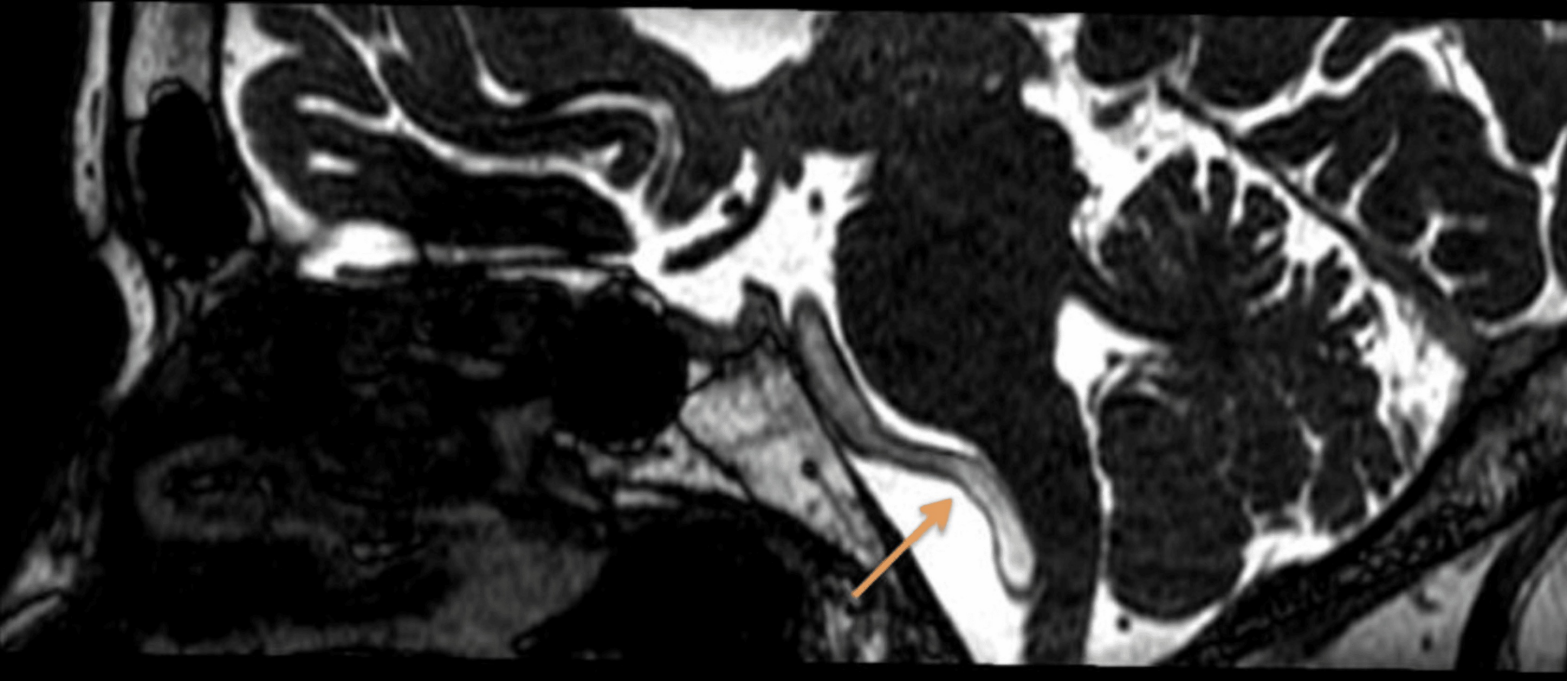
BFFE-sagittal image showing left ectatic vertebral artery compressing the left anterolateral medulla BFFE, balanced fast-field echo

**Figure 6:**
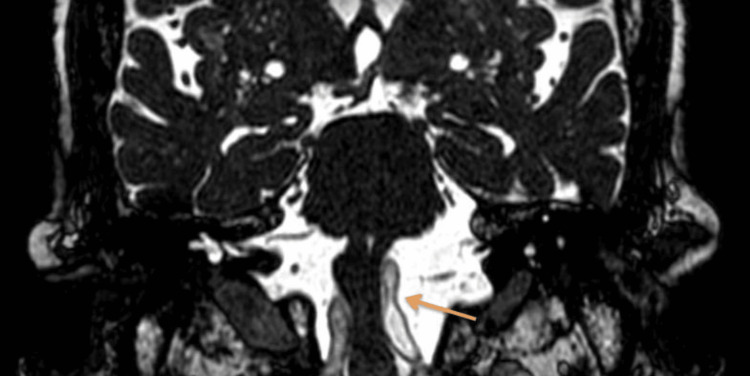
BFFE-coronal image showing left ectatic vertebral artery compressing the left anterolateral medulla BFFE, balanced fast-field echo

The cervical spine MRI demonstrated cervical spondylosis with mild degenerative disc disease at the C5-C6 and C6-C7 levels. There was no evidence of spinal cord compression or significant neural foraminal narrowing. These findings were incidental and not related to the patient’s acute presentation. The brain MRI indicated age-related cerebrocortical atrophy and periventricular hyperintensities consistent with chronic small vessel ischemic changes. T2-weighted images revealed diffuse periventricular and subcortical white matter hyperintensities. FLAIR imaging highlighted these hyperintensities more clearly, while DWI did not show any acute ischemic changes. Crucially, BFFE sequences provided high-resolution images showing significant dolichoectasia of the left vertebral artery, which appeared tortuous and elongated, causing marked compression on the left anterolateral aspect of the medulla oblongata. Figure 1 shows the BFFE sequence of the MRI brain, demonstrating the ectatic vessel and compression of the medulla. Figure 2 shows the MRA sequence and Figures 3, 4 show T1 and T2 sequences with similar findings. Figures 5, 6 show the BFFE sequence of the MRI brain in sagittal and coronal sections, respectively. The imaging findings correlated well with the clinical presentation. The recurrent falls and transient loss of consciousness were attributed to the compression of the left anterolateral medulla by the dolichoectatic left vertebral artery. This compression likely disrupted autonomic and motor pathways, leading to the observed symptoms.

Due to the significant compression and the patient's symptoms, a multidisciplinary approach involving neurosurgery, neurology, and interventional radiology was taken. The management plan included optimizing antihypertensive therapy and implementing fall prevention strategies as immediate conservative measures. Given the severity of symptoms and imaging findings, the patient was considered for potential endovascular interventions, such as stent placement or balloon angioplasty, to relieve the compression. Additionally, a neurosurgical consultation was sought to discuss the feasibility of decompressive surgery if endovascular measures were deemed insufficient or inappropriate. However, given the patient's advanced age and the risks associated with surgical and endovascular procedures, a conservative management plan was deemed most appropriate. This decision was based on the patient's overall health status, potential procedural risks, and the need to minimize invasive interventions. Regular follow-up and monitoring were recommended to assess the patient's response to conservative treatment and to detect any progression of symptoms. The emphasis was placed on managing blood pressure effectively and ensuring safety measures to prevent falls. This conservative approach aims to balance symptom relief with minimizing procedural risks, particularly in elderly patients with multiple comorbidities. This case underscores the importance of advanced imaging techniques in diagnosing complex neurovascular conditions. The use of high-resolution BFFE sequences was pivotal in identifying the ectatic vertebral artery and its compressive effects on the medulla. Early and accurate diagnosis through advanced imaging modalities is crucial for guiding appropriate management and improving patient outcomes. This comprehensive report highlights the need for a multidisciplinary approach in managing such cases to ensure optimal patient care.

## Discussion

Vertebral artery dolichoectasia, marked by elongation, dilation, and tortuosity of the vertebral arteries, can have significant clinical implications, particularly when it compresses adjacent neural structures like the medulla oblongata. The medulla, a vital part of the brainstem, contains essential autonomic and motor pathways, and its compression can lead to various neurological symptoms, including recurrent falls and transient loss of consciousness. In this case, advanced imaging using MRI was instrumental in diagnosing the underlying cause of the patient’s symptoms.

In an elderly hypertensive patient presenting with recurrent falls and transient loss of consciousness, differential diagnoses included TIAs and other cerebrovascular incidents. Initial tests, including echocardiography and a four-vessel Doppler study, showed no abnormalities, prompting further investigation with an MRI of the cervical spine and brain. This imaging provided essential details about the patient's condition.

The MRI of the cervical spine showed cervical spondylosis with mild degenerative disc disease, which were incidental findings and not directly related to the patient's acute symptoms. More importantly, the brain MRI, utilizing high-resolution BFFE sequences, identified significant dolichoectasia of the left vertebral artery. This ectatic artery was exerting marked compression on the left anterolateral aspect of the medulla oblongata. The use of BFFE sequences was particularly valuable in this context, as these sequences provided detailed visualization of the neurovascular structures, enabling precise identification of the neurovascular conflict.

Dolichoectasia of the vertebral arteries can be attributed to several etiologies as explained in Table 1, including chronic hypertension, atherosclerosis, connective tissue disorders, aging, genetic predisposition, inflammatory vasculopathies, and congenital anomalies. In the present case, chronic hypertension likely played a significant role in the development of dolichoectasia. Persistent high blood pressure can lead to increased hemodynamic stress on the arterial walls, resulting in their elongation and dilation over time [1,2].

**Table 1:** Causes of vertebral artery dolichoectasia

Cause	Description	Mechanism
Chronic Hypertension	Persistent high blood pressure leads to increased hemodynamic stress on the arterial walls, causing elongation and dilation.	Hypertensive vascular remodeling and weakening of arterial walls.
Atherosclerosis	Accumulation of lipid plaques within the arterial walls results in vessel rigidity and tortuosity.	Chronic inflammatory response and endothelial dysfunction leading to arterial wall thickening and loss of elasticity.
Connective Tissue Disorders	Conditions such as Ehlers-Danlos syndrome and Marfan syndrome affect the structural integrity of the arterial walls.	Genetic mutations resulting in defective collagen and elastin, leading to weakened arterial walls.
Aging	Natural degenerative changes in the arterial walls with advancing age contribute to vessel elongation and tortuosity.	Decreased elasticity and increased rigidity of arterial walls due to cumulative oxidative stress and degeneration of connective tissue.
Genetic Predisposition	Inherited genetic factors that predispose individuals to vascular abnormalities.	Specific gene mutations affecting vascular development and maintenance.
Inflammatory Vasculopathies	Conditions such as giant cell arteritis and Takayasu arteritis cause inflammation and damage to the arterial walls.	Chronic inflammation leading to granulomatous lesions, fibrosis, and vascular remodeling.
Congenital Anomalies	Developmental anomalies during embryogenesis can result in abnormal vessel morphology.	Disruption in normal vascular development leading to malformed and tortuous arteries.

The compression of the left anterolateral medulla by the dolichoectatic left vertebral artery likely disrupted vital autonomic and motor pathways, leading to the patient’s recurrent falls and transient loss of consciousness. The medulla oblongata is responsible for regulating essential functions such as cardiovascular and respiratory control, as well as motor coordination and balance. Compression in this region can, therefore, lead to significant clinical manifestations, as observed in the current case [3].

Management of vertebral artery dolichoectasia causing medullary compression requires a multidisciplinary approach, involving neurosurgery, neurology, and interventional radiology. Conservative treatment may include antiplatelet or anticoagulant therapy to prevent ischemic events, while surgical options such as microvascular decompression are considered in cases where symptoms are severe or progressive. Endovascular interventions, including stent placement, may be employed in select cases to stabilize the vessel and alleviate compression. This multidisciplinary approach ensures that treatment is tailored to the patient's clinical presentation and imaging findings, optimizing outcomes and minimizing complications​.

Review of literature on vertebral artery dolichoectasia

A review of the literature on vertebral artery dolichoectasia is discussed in Table 2.

**Table 2:** Review of literature on vertebral artery dolichoectasia TIAs, transient ischemic attacks

Study	Objective	Methods	Key Findings	Conclusion
Mohammad Reza Najafi, Nafiseh Toghianifar, Morteza Abdar Esfahani, et al. (2016) [1]	To investigate the prevalence and risk factors of vertebrobasilar dolichoectasia in patients with ischemic stroke and TIAs.	Retrospective analysis of 150 patients with ischemic stroke or TIAs. Clinical data and imaging studies were reviewed.	Vertebrobasilar dolichoectasia was found in 12% of patients. Chronic hypertension and atherosclerosis were identified as significant risk factors.	Vertebrobasilar dolichoectasia is relatively common in patients with ischemic stroke or TIAs, with chronic hypertension being a major contributing factor.
Amarenco P, Hauw JJ (1990) [2]	To describe the clinical and pathological features of cerebellar infarction in the territory of the anterior and inferior cerebellar artery.	Case series of 20 patients with cerebellar infarction. Clinical evaluations and post-mortem examinations were conducted.	Cerebellar infarctions were often associated with vertebral artery dolichoectasia. Symptoms included vertigo, ataxia, and dysarthria.	Vertebral artery dolichoectasia can lead to significant neurological deficits due to its impact on the posterior circulation.
Jacques M Conradie, Embrensia G Bonnet (2021) [3]	To assess the prevalence of dolichoectasia of intracranial arteries in a multiethnic population.	Cross-sectional study involving 500 participants from diverse ethnic backgrounds. MRI was used for vascular assessment.	Dolichoectasia was present in 8% of the population, with a higher prevalence in older adults and those with hypertension.	Dolichoectasia of intracranial arteries is a notable finding in the elderly and hypertensive populations, necessitating careful monitoring and management.
X Wu, Y Xu, B Hong, et al. (2013) [4]	To evaluate the outcomes of surgical and endovascular treatments for vertebrobasilar dolichoectasia.	Prospective study of 50 patients undergoing surgical or endovascular treatment. Follow-up for two years to assess outcomes.	Both surgical and endovascular treatments were effective in reducing symptoms and preventing recurrence. Surgical intervention had a higher complication rate.	Treatment of vertebrobasilar dolichoectasia requires careful selection of patients for surgical vs. endovascular approaches to minimize complications and optimize outcomes.
Feng Wan, Xiao Yun Hu, Tao Wang, et al. (2018) [5]	To investigate the clinical and radiologic characteristics of patients with vertebrobasilar dolichoectasia.	Retrospective review of 100 patients with vertebrobasilar dolichoectasia. Clinical presentations and imaging features were analyzed.	Common symptoms included headaches, dizziness, and cranial nerve palsies. MRI findings correlated well with clinical presentations.	Vertebrobasilar dolichoectasia presents with diverse clinical symptoms, and MRI is crucial for accurate diagnosis and management planning.
Jiejun Wang, Luqiong Jia, Xinjian Yang, et al. (2019) [6]	To systematically review the safety and efficacy of endovascular treatment for vertebrobasilar dolichoectasia.	Meta-analysis of 20 studies involving endovascular treatments. Data on efficacy, safety, and complications were pooled and analyzed.	Endovascular treatment was effective in symptom relief and vascular remodeling, with a lower complication rate compared to surgical approaches.	Endovascular intervention is a promising option for managing vertebrobasilar dolichoectasia, with favorable safety and efficacy profiles.
Xu SY, Wang RJ, Zhang L, et al. (2021) [7]	To examine the genetic factors contributing to vertebrobasilar dolichoectasia.	Genetic analysis of 200 patients with vertebrobasilar dolichoectasia and 200 controls. Whole-exome sequencing was performed.	Mutations in genes related to connective tissue integrity, such as COL4A1 and TGFBR2, were more prevalent in patients.	Genetic predisposition plays a significant role in the development of vertebrobasilar dolichoectasia, indicating the need for genetic screening in at-risk populations.

## Conclusions

Vertebral artery dolichoectasia, especially when causing compression of the medulla oblongata, presents a significant clinical challenge due to its complex presentation and potential for serious neurological symptoms. This condition, often associated with chronic hypertension, atherosclerosis, and other underlying factors, requires a nuanced approach to diagnosis and management. Advanced imaging techniques such as MRI are crucial for accurately identifying and characterizing the dolichoectatic vessels and their impact on adjacent neural structures.

The reviewed literature underscores the multifactorial etiology of vertebral artery dolichoectasia and highlights the effectiveness of both surgical and endovascular treatments, with careful patient selection being paramount. Genetic predispositions also play a notable role, suggesting the need for broader screening in at-risk populations. The present case report and the supporting literature emphasize the importance of a multidisciplinary approach in managing vertebral artery dolichoectasia, aiming to optimize patient outcomes through precise diagnosis, targeted therapy, and comprehensive care strategies.
